# Holistic dementia care continuum from the point of diagnosis to the end of life: Progress and challenges in translating national dementia policies into daily practice in Japan

**DOI:** 10.1002/alz.086501

**Published:** 2025-01-09

**Authors:** Miharu Nakanishi, Asao Ogawa, Atsushi Nishida

**Affiliations:** ^1^ Tohoku University Graduate School of Medicine, Sendai‐shi, Miyagi Japan; ^2^ Leiden University Medical Center, Leiden, Zuid‐Holland Netherlands; ^3^ Tokyo Metropolitan Institute of Medical Science, Setagaya‐ku, Tokyo Japan; ^4^ Division of Psycho‐Oncology, Exploratory Oncology Research and Clinical Trial Center, National Cancer Center, Kashiwa, Chiba Japan

## Abstract

**Background:**

The WHO’s Global Dementia Action Plan comprises seven action areas, including dementia diagnosis, treatment, care, and support. Palliative care is called for as a core component of the care continuum from the diagnosis to the end of life. Japan has pursued a holistic care approach in dementia policies. However, from our review, we found that palliative care components are not fully represented. We aim to present how Japanese policies have been translated into practice and highlight challenges that remain to be addressed.

**Method:**

We performed empirical research derived from real‐world data to examine the potential gaps between policies and daily practice in Japan. We focused on four topics: advance care planning, neuropsychiatric symptoms, physical restraint in acute care, and end‐of‐life care.

**Result:**

Engagement of advance care planning was typically triggered by a clinical dementia diagnosis, and the doctor providing the diagnosis was not often involved in the conversation with the patient. We developed a psychosocial intervention program (DEMBASE®) to reduce neuropsychiatric symptoms in collaboration with a Swedish BPSD‐registry team, for which effectiveness was verified. Physical restraint of inpatients was prevalent even in units with a dementia care benefit. The onset of the COVID‐19 pandemic triggered the shift to hospital deaths, which were uniquely observed in deaths from dementia.

**Conclusion:**

Japanese dementia policies promote “aging in place,” which is now a mainstream strategy globally. However, dementia care remains suboptimal. Baseline palliative care should be integrated into dementia diagnoses, treatment, care, and support.

